# iview: an interactive WebGL visualizer for protein-ligand complex

**DOI:** 10.1186/1471-2105-15-56

**Published:** 2014-02-25

**Authors:** Hongjian Li, Kwong-Sak Leung, Takanori Nakane, Man-Hon Wong

**Affiliations:** 1 Department of Computer Science and Engineering, Chinese University of Hong Kong, Hong Kong, China; 2 Graduate School of Medicine, Kyoto University, Kyoto, Japan

**Keywords:** Structural bioinformatics, Visualization, Molecular docking

## Abstract

**Background:**

Visualization of protein-ligand complex plays an important role in elaborating protein-ligand interactions and aiding novel drug design. Most existing web visualizers either rely on slow software rendering, or lack virtual reality support. The vital feature of macromolecular surface construction is also unavailable.

**Results:**

We have developed iview, an easy-to-use interactive WebGL visualizer of protein-ligand complex. It exploits hardware acceleration rather than software rendering. It features three special effects in virtual reality settings, namely anaglyph, parallax barrier and oculus rift, resulting in visually appealing identification of intermolecular interactions. It supports four surface representations including Van der Waals surface, solvent excluded surface, solvent accessible surface and molecular surface. Moreover, based on the feature-rich version of iview, we have also developed a neat and tailor-made version specifically for our istar web platform for protein-ligand docking purpose. This demonstrates the excellent portability of iview.

**Conclusions:**

Using innovative 3D techniques, we provide a user friendly visualizer that is not intended to compete with professional visualizers, but to enable easy accessibility and platform independence.

## Background

Visualization of protein-ligand complex plays an important role in elaborating protein-ligand interactions and aiding novel drug design. To date, dozens of visualization tools already exist. VMD [1], PyMOL (http://www.pymol.org) and Chimera [2] are very well-known and highly cited. They can interpret multiple file formats and generate multiple representations to supply precise and powerful control. AutoDockTools4 [3] provides native support for the PDBQT file format, which is widely used in various protein-ligand docking software such as AutoDock [3], AutoDock Vina [4], and our idock [5]. We also developed our own method [6] to visualize structures in virtual reality settings and employ fragment-based *de novo* ligand design strategy for interactive drug design. PoseView [7] and LigPlot+ [8], on the other hand, plot 2D diagrams of protein-ligand interactions from 3D coordinates.

In addition, there are web visualizers based on either Java applet, Adobe Flash, or HTML5 canvas. Jmol (http://www.jmol.org), an open source Java viewer for chemical structures in 3D, has been deployed worldwide and recognized as the *de facto* molecular viewer on the web. GIANT [9], a web visualizer based on Jmol, supports analyzing protein-ligand interactions on the basis of patterns of atomic contacts obtained from the statistical analyses of 3D structures. However, Java is being disabled on more and more systems due to security concerns so that Java-free visualizers are highly required. JSmol [10], a JavaScript-only version of Jmol, includes the full implementation of the entire set of Jmol functionalities. Although Jmol and JSmol support a large set of advanced features including scripting, they rely on software rendering which is slow on large display areas and thus prevents detailed inspection of the structure. In contrast, WebGL visualizers benefit from GPU acceleration. For instance, ChemDoodle Web Components (http://web.chemdoodle.com), a pure JavaScript chemical graphics and cheminformatics library, presents 2D and 3D graphics and animations for chemical structures, reactions and spectra, but it lacks protein surface construction. GLmol (http://webglmol.sourceforge.jp), a molecular viewer on WebGL/JavaScript using the three.js library, supports multiple file formats and representations, and features an experimental version of surface construction based on the EDTSurf algorithm [11,12]. Another study [13] also presents a WebGL technology for rendering molecular surface using the SpiderGL library [14]. Nevertheless, none of these WebGL visualizers support virtual reality effects.

Surface representation is a convenient way to visualize protein-ligand interactions. However, macromolecular surface calculation is computationally and memory intensive. Furthermore, the calculated mesh is very complex, often exceeding 500,000 polygons. Therefore its implementation in JavaScript/WebGL was considered to be very difficult. Most existing web visualizers either rely on slow software rendering, or lack virtual reality support. Moreover, the vital feature of protein surface construction is usually unavailable, and the support for PDBQT format is not implemented.

To address the above obstacles, we have developed iview, an interactive WebGL visualizer of protein-ligand complex, featuring three special effects in virtual reality settings and four surface representations (Table 1). Furthermore, we show that iview can be easily modified to adapt to different applications. As an application example, we have recently developed a web platform called istar [15] to automate large-scale protein-ligand docking using our idock [5]. Refactored from the feature-rich version of iview, we have also developed tailor-made version specifically for visualizing docking input data and output results of user-submitted jobs.

**Table 1:** Full features of iview

**Category**	**Features**
File format input	PDB
	PDBQT
Camera	perspective
	orthographic
Background	black
	grey
	white
Structure coloring	atom spectrum
	protein chain
	protein secondary structure
	B factor
	residue name
	residue polarity
	atom type
Primary structure	line
	stick
	ball & stick
	sphere
	dot
Secondary structure	ribbon
	strand
	cylinder & plate
	C alpha trace
	B factor tube
Protein surface	Van der Waals surface
	solvent excluded surface
	solvent accessible surface
	molecular surface
Proteins surface opacity	1.0
	0.9
	0.8
	0.7
	0.6
	0.5
Protein surface wireframe	yes
	no
Atom and residue labeling	yes
	no
Virtual reality effect	anaglyph
	parallax barrier
	oculus rift
Canvas manipulation	mouse
	hand touch
Manipulation mode	rotation
	translation
	zooming
	slab
Canvas export	png

iview is the only web visualizer that is accelerated by GPU hardware and supports three unique features: protein surface construction, virtual reality effects, and PDBQT format input.

## Implementation

iview is refactored from GLmol 0.47, using three.js as its primary 3D engine with antialiasing support. It is based on WebGL canvas and can be easily integrated into existing HTML5 web pages to display molecular models without requiring Java or browser plugins. It loads a protein-ligand structure from the PDB (Protein Data Bank) [16] as its data source via a RESTful interface. It renders four standard representations of primary structure, namely line, stick, ball & stick and sphere, and five standard representations of secondary structure, namely ribbon, strand, cylinder & plate, C alpha trace and B factor tube. It colors the structure by either atom spectrum, protein chain, protein secondary structure, B factor, residue name, residue polarity, or atom type, by setting the vertex colors of the geometry object of the corresponding representation. It supports user interactions including rotation, translation, zooming and slab with mouse or hand touch manipulation. It provides both perspective and orthographic cameras, and anaglyph, parallax barrier and oculus rift effects from three.js examples for use in a virtual reality environment.

We have ported EDTSurf [11,12], an fast algorithm to generating triangulated macromolecular surfaces by Euclidean distance transform, to JavaScript and integrated it into iview to construct and render in real time four representations of protein surface, namely Van der Waals surface, solvent excluded surface, solvent accessible surface and molecular surface, with opacity and wireframe adjustable by users. Note that molecular surface is in fact solvent excluded surface, but EDTSurf uses different ways to derive them. We therefore provide them both as two different surface representations in iview. Although the JavaScript implementation of the EDTSurf algorithm typically consumes a few seconds and 500MB to 700MB memory for computation, it is sufficiently efficient for practical applications. To limit CPU and memory usage, the calculation grid size is restricted to 180×180×180.

It is worthwhile to highlight that iview performs all parsing and rendering in the client browser, without any dependency on server side at all, ensuring the data privacy is maintained. This is unlike ChemDoodle Web Components, some of whose functions send data to a dedicated server for processing and wait for retrieval of results.

The differences between iview and GLmol are listed in the Additional file 1.

## Results

We take as example the CCR5 chemokine receptor-HIV entry inhibitor maraviroc complex [17] (PDB code: 4MBS).

**Figure 1:**
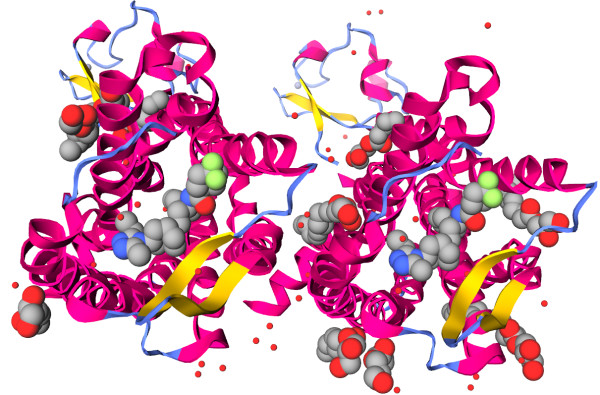
**iview rendering of the CCR5 chemokine receptor-HIV entry inhibitor maraviroc complex **[17]** (PDB code: 4MBS).** The secondary structure of human CCR5 is rendered as ribbon. The marketed HIV drug maraviroc is rendered as sphere. This figure can be reproduced at http: //istar.cse.cuhk.edu.hk/iview/?4MBS.

Figure 1 shows the human CCR5 secondary structure rendered as ribbon, and the ligands rendered as sphere. 

**Figure 2:**
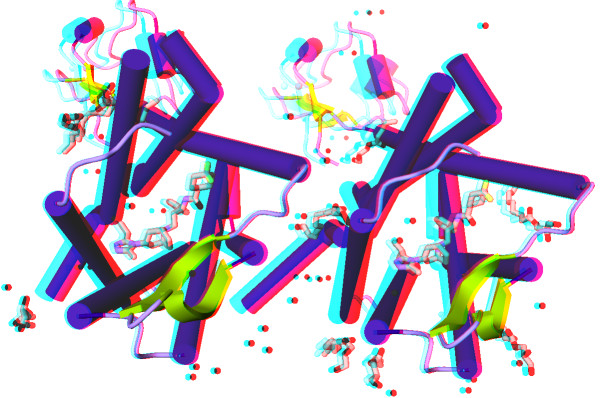
**iview rendering of the CCR5 chemokine receptor-HIV entry inhibitor maraviroc complex **[17]** (PDB code: 4MBS), with anaglyph effect enabled.** The anaglyph effect encodes each eye’s image using filters of chromatically opposite colors to achieve stereoscopic 3D effect. When users wear a spectacle with special filters on both sides, each of the two differently filtered colored images reaches one eye, revealing an integrated stereoscopic image. This figure can be reproduced at http://istar.cse.cuhk.edu.hk/iview/?4MBS.

Figure 2 shows the anaglyph effect in a virtual reality environment. When users wear a spectacle with special filters on both sides, the disparity between two superimposed molecules creates a perception of depth, leading to visually more appealing identification of intermolecular interactions. The parallax barrier and oculus rift effects are illustrated in the Additional files 2 and 3. 

**Figure 3:**
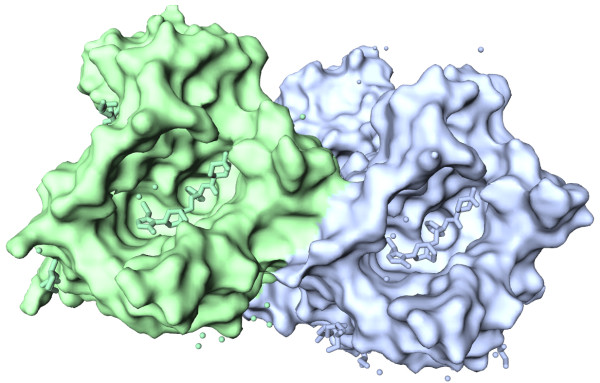
**iview rendering of the CCR5 chemokine receptor-HIV entry inhibitor maraviroc complex **[17]** (PDB code: 4MBS), with protein surface enabled.** The human CCR5 is rendered as molecular surface colored by chain. The marketed HIV drug maraviroc is rendered as stick colored by chain. It can be clearly seen that the crystal structure consists of two complexes, and the CCR5 forms a deep allosteric cavity where maraviroc is buried. This figure can be reproduced at http://istar.cse.cuhk.edu.hk/iview/?4MBS.

Figure 3 shows the protein surface generated by our JavaScript implementation of the EDTSurf algorithm [11,12]. The human CCR5 is rendered as molecular surface colored by chain. The marketed HIV drug maraviroc is rendered as stick colored by chain. It can be clearly seen that the asymmetric unit is composed of two complexes, and the CCR5 forms a deep allosteric cavity where maraviroc binds.

We have successfully tested iview in Chrome 30, Firefox 25, Safari 6.1 and Opera 17. Support for IE 11 is experimental because gl_FrontFacing is unsupported in IE 11. Refer to http://caniuse.com/webgl, for compatibility of WebGL support in desktop and mobile browsers.

## Application example

We emphasize portability and usability, and illustrate that iview can be easily modified to suit one’s particular application, given that iview is free and open source under a permissive license. We take protein-ligand docking as an example. Based on the feature-rich version of iview, our tailor-made version specifically for idock jobs cleans up many dispensable functions, enabling a very neat interface. It only retains the rendering of primary structure of protein and ligand, and the construction of protein surface. Most importantly, it implements new features especially for protein-ligand docking purpose.

In the input phase of a docking job, it merely requires a PDB file, which can be obtained either from the PDB database [16] or via homology modeling, and then constructs the protein surface asynchronously in a separate web worker to keep the web page responsive. It automatically detects a binding site from the largest co-crystallized ligand first by finding the smallest cubic box that covers the entire ligand and then by extending the box by 50% in all the three dimensions in order to reserve space for conformational sampling. In case of non-existence of co-crystallized ligand, the binding site is defaulted to the geometric center of the protein. The binding site is visually depicted in the form of a cubic box whose center and size can be manually adjusted by users in real time.

**Figure 4:**
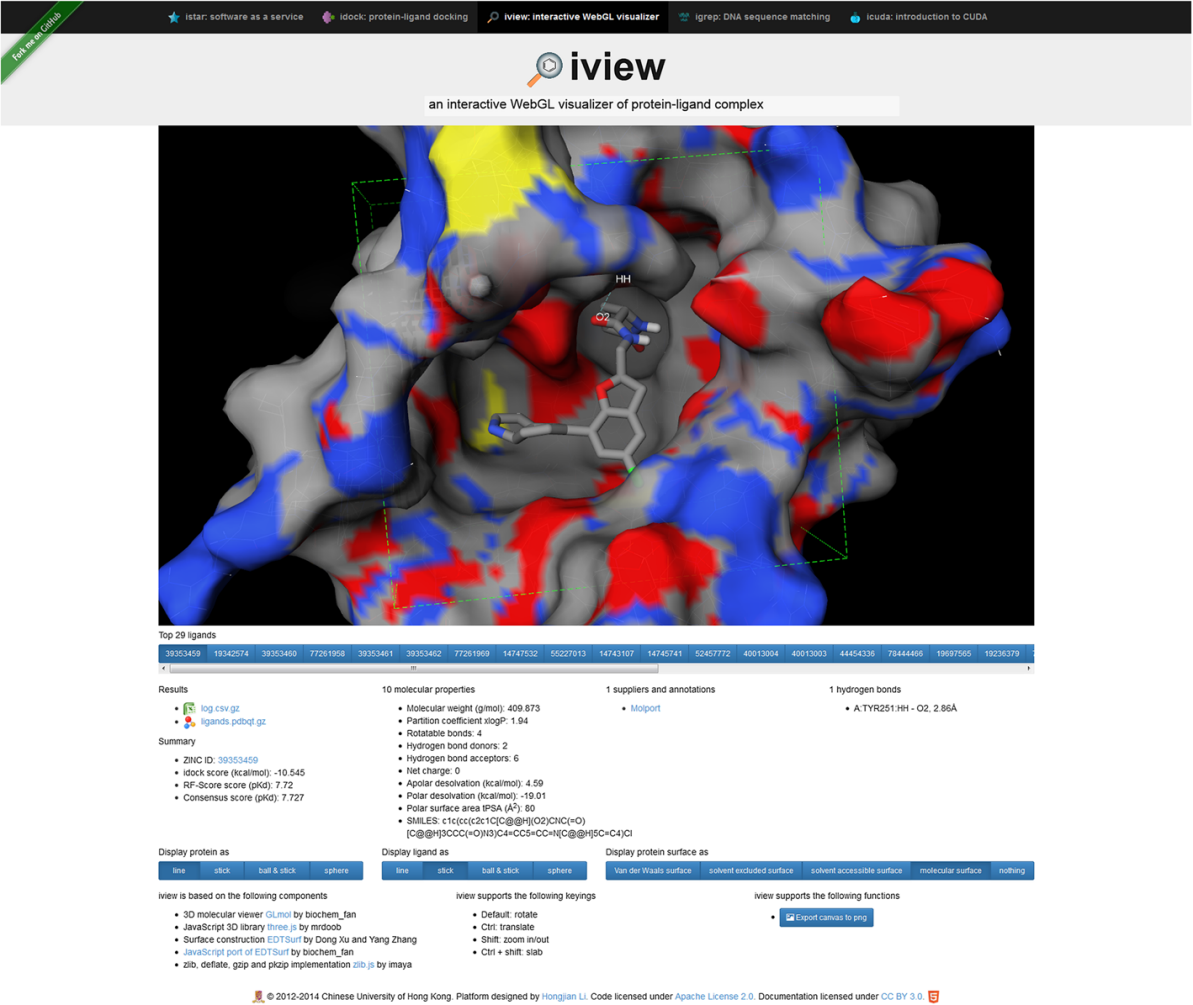
**Tailor-made version of iview specifically for visualizing docking results of user-submitted jobs.** It displays the user-supplied search space in the form of a cubic box so that the binding site is visually depicted. It displays the top hit ligand IDs in a horizontally scrollable row and provides a straightforward way to switch ligands easily. It lists the docking result files, predicted binding affinity values, molecular properties, compound suppliers and annotations, and putative hydrogen bonds, in order to give users a quick overview of the top hit ligands, and assist them in making decisions of which compounds to purchase for subsequent wet-lab experiments. This figure can be reproduced at http://istar.cse.cuhk.edu.hk/idock/iview/?525a0abab0717fe31a000001.

In the output phase of a docking job, it displays the user-supplied cubic box for users to confirm that the predicted ligand conformations do fall inside the desired binding site. Other than PDB format, its parsers are capable of parsing a protein and multiple top hit ligands in PDBQT format used by idock. It displays the top hit ligand IDs in a horizontally scrollable row and provides a straightforward way to switch ligands easily through a button group. It has built-in support for putative intermolecular hydrogen bond detection by finding hydrogen bond donors and acceptors from protein and ligand and setting the distance threshold to 3.5Å. It automatically annotates important atoms, like those involving in intermolecular hydrogen bonds, by placing labels next to the corresponding atoms in the canvas. It lists the docking result files, predicted free energy and binding affinity values, molecular properties, SMILES representation, compound suppliers and annotations, and putative hydrogen bond positions and their lengths, in order to give users a quick overview of the top hit ligands and assist them in making decisions of which compounds to purchase for subsequent wet-lab experiments (Figure 4).

## Conclusions

We have designed and developed iview to be a simple and straightforward way to visualize protein-ligand complex. It enables non-experts to quickly elucidate protein-ligand interactions in a 3D manner. Furthermore, iview is free and open source, and can be easily integrated into any bioinformatics application that requires interactive protein-ligand visualization.

## Availability and requirements

**Project name:** iview

**Project home page:**http://istar.cse.cuhk.edu.hk/iview,

**Operating system:** Platform independent

**Programming languages**: JavaScript, HTML5, CSS3

**Other requirements:** Browser and graphics card with WebGL capability

**License:** Apache License 2.0

## Competing interests

The authors declare that they have no competing interests.

## Authors’ contributions

HL and TN developed the presented software. HL drafted the manuscript. TN, KWL and MHW edited the manuscript. All authors read and approved the final manuscript.

## Supplementary Material

Additional file 1 **Differences between iview and GLmol.** The text file lists the differences between iview and GLmol. Click here for file

Additional file 2 **iview rendering of the CCR5 chemokine receptor-HIV entry inhibitor maraviroc complex **[17]** (PDB code: 4MBS), with parallax barrier effect enabled.** A parallax barrier is a device placed in front of a LCD (Liquid Crystal Display) to permit a stereoscopic or multiscopic image without 3D glasses. The device is composed of a layer of material with precision slits, enabling each eye to see a different set of pixels and thus creating a sense of depth through parallax. Click here for file

Additional file 3 **iview rendering of the CCR5 chemokine receptor-HIV entry inhibitor maraviroc complex **[17]** (PDB code: 4MBS), with oculus rift effect enabled.** The Oculus Rift is a virtual reality head-mounted device, which features a high-speed inertial measurement unit and a LCD display, visible via dual lenses positioned over the eyes to provide a 90 degrees horizontal and 110 degrees vertical stereoscopic 3D perspective. Click here for file
