# Supplementary figures and images for: iview: an interactive WebGL visualizer for protein-ligand complex
Source: BMC Bioinformatics. 2014 Feb 25;15:56. doi: 10.1186/1471-2105-15-56 (PMC3936933; doi:10.1186/1471-2105-15-56)

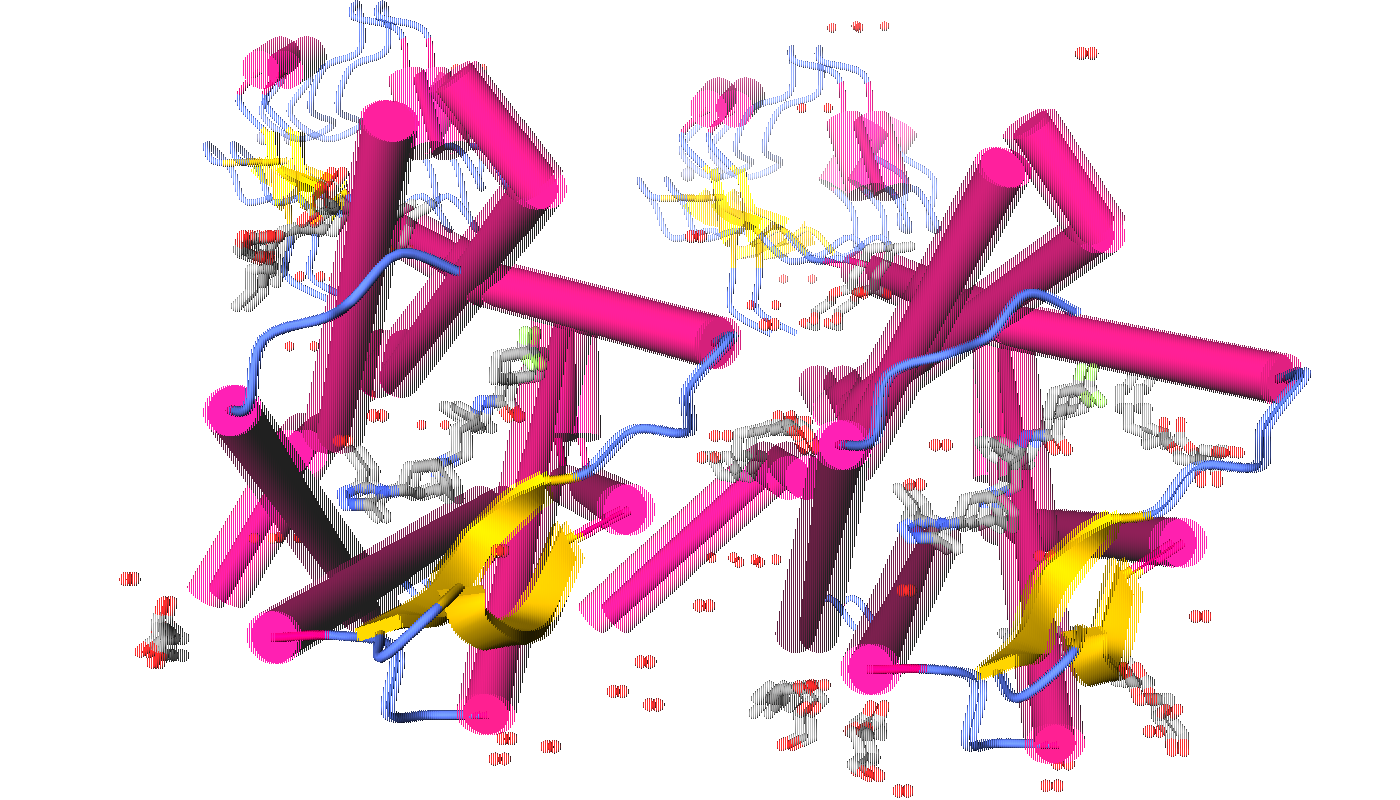

Supplement: Additional file 2 — iview rendering of the CCR5 chemokine receptor-HIV entry inhibitor maraviroc complex [17] (PDB code: 4MBS), with parallax barrier effect enabled. A parallax barrier is a device placed in front of a LCD (Liquid Crystal Display) to permit a stereoscopic or multiscopic image without 3D glasses. The device is composed of a layer of material with precision slits, enabling each eye to see a different set of pixels and thus creating a sense of depth through parallax. [file 1471-2105-15-56-S2.png]

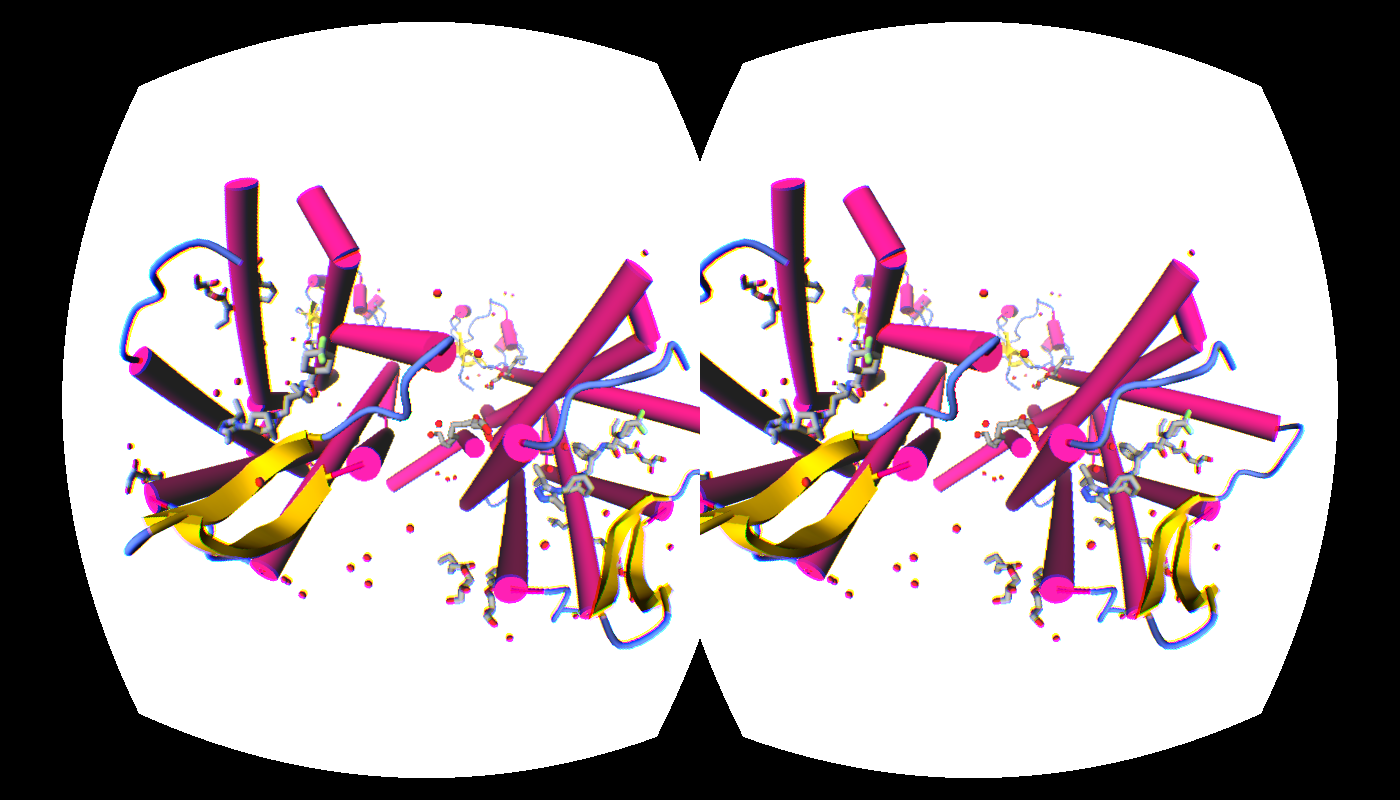

Supplement: Additional file 3 — iview rendering of the CCR5 chemokine receptor-HIV entry inhibitor maraviroc complex [17] (PDB code: 4MBS), with oculus rift effect enabled. The Oculus Rift is a virtual reality head-mounted device, which features a high-speed inertial measurement unit and a LCD display, visible via dual lenses positioned over the eyes to provide a 90 degrees horizontal and 110 degrees vertical stereoscopic 3D perspective. [file 1471-2105-15-56-S3.png]
